# Targeting miR‐193a‐AML1‐ETO‐β‐catenin axis by melatonin suppresses the self‐renewal of leukaemia stem cells in leukaemia with t (8;21) translocation

**DOI:** 10.1111/jcmm.14399

**Published:** 2019-05-22

**Authors:** Bin Zhou, Haige Ye, Chongyun Xing, Bin Liang, Haiying Li, Linling Chen, Xingzhou Huang, Yanfei Wu, Shenmeng Gao

**Affiliations:** ^1^ Laboratory of Internal Medicine The First Affiliated Hospital of Wenzhou Medical University Wenzhou China; ^2^ Department of Hematology The First Affiliated Hospital of Wenzhou Medical University Wenzhou China; ^3^ Department of Clinical Laboratory The People's Hospital of Yuhuang County Taizhou China

**Keywords:** AML1‐ETO, leukaemia stem cell, melatonin, miR‐193a, self‐renewal

## Abstract

AML1‐ETO, the most common fusion oncoprotein by t (8;21) in acute myeloid leukaemia (AML), enhances hematopoietic self‐renewal and leukemogenesis. However, currently no specific therapies have been reported for t (8;21) AML patients as AML1‐ETO is still intractable as a pharmacological target. For this purpose, leukaemia cells and AML1‐ETO‐induced murine leukaemia model were used to investigate the degradation of AML1‐ETO by melatonin (MLT), synthesized and secreted by the pineal gland. MLT remarkedly decreased AML1‐ETO protein in leukemic cells. Meanwhile, MLT induced apoptosis, decreased proliferation and reduced colony formation. Furthermore, MLT reduced the expansion of human leukemic cells and extended the overall survival in U937T‐AML1‐ETO‐xenografted NSG mice. Most importantly, MLT reduced the infiltration of leukaemia blasts, decreased the frequency of leukaemia stem cells (LSCs) and prolonged the overall survival in AML1‐ETO‐induced murine leukaemia. Mechanistically, MLT increased the expression of miR‐193a, which inhibited AML1‐ETO expression via targeting its putative binding sites. Furthermore, MLT decreased the expression of β‐catenin, which is required for the self‐renewal of LSC and is the downstream of AML1‐ETO. Thus, MLT presents anti‐self‐renewal of LSC through miR‐193a‐AML1‐ETO‐β‐catenin axis. In conclusion, MLT might be a potential treatment for t (8;21) leukaemia by targeting AML1‐ETO oncoprotein.

## 1 INTRODUCTION

The t (8;21) translocation, which results in acute myeloid leukaemia (AML)1‐ETO fusion gene, is the most frequent translocation in AML. This translocation accounts for about 12%‐15% of de novo AML cases and up to about 40% of AML patients with M2 subtype by French—American—British classification.^1^ Although AML patients with t (8;21) have a comparatively good prognosis and most patients achieve complete remission, almost half of the patients relapse and the 5‐year overall survival rate is only 60%.^2^ This translocation creates an in‐frame fusion gene between the conserved runt homology domain from *AML1* to almost the entire *ETO* gene.^1^ The AML1 encodes a subunit of the core‐binding factor heterodimer, which mediates in transcriptional regulation during hematopoiesis. ETO represses transcription through recruiting a nuclear receptor corepressor, histone deacetylase complex and the mSin3 corepressor.^3^ Thus, AML1‐ETO is believed to block myeloid differentiation via partially inhibiting the transcription of AML1‐driven genes involved in cell differentiation. Multiple studies indicate that AML1‐ETO alone is not sufficient to induce AML in a murine model and thus additional genetic events are required for the onset of AML.^4^ AML1‐ETO rapidly induces murine leukaemia in cooperation with Wilm's tumour‐1 (*WT1*),^5^ suggesting that AML1‐ETO is considered as the important onset oncogene for the leukemogenesis of AML.

Acute myeloid leukaemia is initiated from a small subset of leukaemia stem cell (LSC). Drug‐resistant LSC, which is not completely eradicated by current standard therapies, causes most relapse of AML patients after chemotherapy.^6^ LSC presents several important characteristics, including increased self‐renewal, uncontrolled proliferation and dysregulated differentiation, distinguishing it from normal hematopoietic stem and progenitor cell (HSPC). These characteristics are caused by the expression of some of the leukaemia oncogenes, such as *MLL‐AF9* 7 and *AML1‐ETO*.^8^ AML1‐ETO enhances the self‐renewal capacity of HSPC through COX‐β‐catenin signalling.^9^ Therefore, eliminating AML1‐ETO protein might inhibit the self‐renewal capacity in AML patients with t (8;21). Our previous study has shown that honokiol, a natural phenolic compound isolated from the plant Magnolia officinalis, rapidly degrades AML1‐ETO protein via increasing ubiquitin conjugase UbcH8 expression.^10^ However, whether agents degrading AML1‐ETO protein present anti‐self‐renewal of LSC is not yet determined.

Melatonin (MLT; N‐acetyl‐5‐methoxytryptamine) is an ancient molecule with a wide range of physiological functions.^11^ In mammals, the pineal gland synthesizes and secretes MLT into the blood circulation. However, extrapineal tissues, including bone marrow and skin, might also contribute to MLT levels.^12^ The concentration of MLT may reach up to 0.5 nmol/L in mammalian blood at night. At this physiological concentration, MLT affects the biological clock, regulates the immune system, and presents antioxidant actions.^13^ MLT influences the physiological function mainly through interaction with two well‐characterized G protein‐coupled seven‐transmembrane‐domain receptors, MT1 and MT2, which inhibit adenylate cyclase.^14^ MLT at higher concentration (mM) presents anti‐cancer activity through inhibiting proliferation and inducing apoptosis in multiple types of cancer including leukaemia.^15^ Additionally, MLT enhances the anti‐leukaemia activity of puromycin in HL‐60 cells.^16^ Fusion genes, such as *AML1‐ETO* and *PML‐RARα*, play important roles in leukemogenesis. However, whether MLT presents anti‐leukaemia ability through degrading fusion proteins, such as AML1‐ETO, is not determined. Therefore, we determined whether MLT can degrade AML1‐ETO oncoprotein and found that MLT degraded AML1‐ETO protein and suppressed the self‐renewal of LSCs in leukaemia with positive AML1‐ETO.

In this report, we found that MLT substantially reduces AML1‐ETO protein in leukaemia cell lines, primary AML blasts, and AML1‐ETO‐induced murine leukaemia blasts. MLT induces apoptosis, decreases proliferation and reduces colony formation in leukaemia cells, but not in normal murine and human HSPC cells. Furthermore, MLT prolongs the overall survival in AML1‐ETO‐induced murine leukaemia. In addition, MLT decreases the self‐renewal of LSC via inhibiting the expression of β‐catenin. Mechanistically, MLT decreases AML1‐ETO expression through up‐regulation of miR‐193a. Thus, MLT presents anti‐self‐renewal of LSC through miR‐193a‐AML1‐ETO‐β‐catenin axis in AML cells carrying AML1‐ETO.

## 2 MATERIALS AND METHODS

### 2.1 Leukaemia cell lines, primary AML blasts, mouse lineage‐negative cells and human CD34^+^ cells

Human leukemic cell lines including Kasumi‐1, SKNO‐1, U937, K562 and THP1 (ATCC, Manassas, VA) were purchased for the present study. For SKNO‐1 cells, 10 ng/mL of granulocyte‐macrophage colony‐stimulating (PeproTech, Rocky Hill, NJ) was added. U937‐AML1‐ETO stable transformant (U937‐A/E9/14/18, U937T) was generated as described.^17^ U937T cells were treated with 5 μmol/L ponasterone A (Pon A, Santa Cruz Biotechnology, Santa Cruz, CA) to induce AML1‐ETO expression.^18^ Leukemic cell lines were cultured in RPMI 1640 supplemented with 10% foetal bovine serum (Invitrogen, Carlsbad, CA) and 1% penicillin‐streptomycin in humidified 37°C incubator with 5% CO_2_. Bone marrow mononuclear cells (blasts % >70%) were isolated by Ficoll density gradient centrifugation (GE Healthcare, Uppsala, Sweden) and were obtained from AML patients bearing AML1‐ETO. All studies involving human participants were in accordance with the ethical standards of the Ethics Committee of the First Affiliated Hospital of Wenzhou Medical University and the Declaration of Helsinki. These patients all gave informed consent in accordance with the Declaration of Helsinki. Mononuclear cells isolated from mouse BM were labelled with biotinylated mouse lineage depletion cocktail (Stemcell Technologies, Vancouver, BC, Canada) and were then incubated with streptavidin particles and placed in the magnetic field to remove the lineage‐committed cells from progenitor cells. The lineage‐negative (Lin^−^) fraction of cells was resuspended in Iscove modified Dulbecco medium for colony forming count. Normal human CD34^+^ cells were isolated and enriched from umbilical cord blood by positive selection (Stemcell Technologies).

### 2.2 mRNA extract and quantitative real‐time PCR

Total RNA was extracted by TRIzol (Invitrogen) and reverse transcription (RT) was performed by Fermentas retrovirus kit (Thermo Scientific, Waltham, MA) according to the manufacturer's instructions. Quantitative real‐time PCR (qRT‐PCR) was performed using SYBR Green PCR Master Mix (Qiagen). GAPDH was used for the endogenous control for the transcriptional expression of *Bcl‐2*, myeloperoxidase (*MPO*) and others. Mature miR‐193a and U6 snRNA were reversely transcribed using stem‐loop RT primer with miscript Ⅱ RT Kit (Qiagen). Expression data were uniformly normalized to the endogenous control U6. Relative expression was calculated using the 2^−ΔΔCT^ method. The primers of AML1‐ETO were selected according to previous reports^19^, ^20^ and primers of other gene transcripts were shown in Table S1.

### 2.3 Construction of plasmids

To construct the plasmid expressing miR‐193a in mammalian cells, the paired primers were based on the primary sequence of pre‐miR‐193a and its flanking regions. Then, the PCR product was cloned into lentiviral expression vector pLVX‐IRES‐ZsGreen1 (pLVX‐GFP, Clontech, Palo Alto, CA). The coding sequence of β‐catenin was constructed in pLVX‐GFP to produce plasmid expressing human β‐catenin. MSCV‐GFP‐IRES‐AML1‐ETO was kindly provided by Prof. Ying Lu (Department of Pathophysiology, Shanghai Jiao Tong University School of Medicine). Whole CDS of WT1 was directly synthesized (Genewiz, Suzhou, China) and then inserted into retrovirus vector pMSCV‐puro (Clontech).^21^ All plasmids were confirmed by sequencing.

### 2.4 Limiting dilution assays

GFP^+^BM leukemic cells (sorted by flow cytometry) were collected from secondary BMT recipients. Three different doses of donor cells were transplanted into lethally irradiated recipients for each group. The numbers of recipient mice were counted only when they developed full‐blown leukaemia within 20 weeks post‐transplant. Extreme limiting dilution assay software^22^ was used to determine the frequency of LSC.2.5. Inhibition of miR‐193a expression by Agomir in leukemic cells.

Leukemic cells were seeded in 6‐well plates at a density of 2.0 × 10^5^/mL per well. Special miR‐193a inhibitor Agomir (100 pmol per well, GenePharma, Shanghai, China) or negative control was transiently transfected into leukemic cells by Hiperfect transfection reagent (Qiagen). Cells were collected and proteins were extracted for western blot after transfection for 48 hours.

### 2.5 Other procedures

For details on reagents, apoptosis detection, virus production and cell transduction, western blot, luciferase reporter and mutagenesis assays, and other experiments sees supplemental materials and methods.

### 2.6 Statistical analysis

All the results were expressed as Mean ± SD where applicable. The significance of the difference between groups was determined by Student's *t* test. A *P* < 0.05 was considered statistically significant. All statistical analyses were performed with SPSS software (SPSS 22.0, Chicago, IL).

## 3 RESULTS

### 3.1 Degradation of AML1‐ETO by MLT

**Figure 1:**
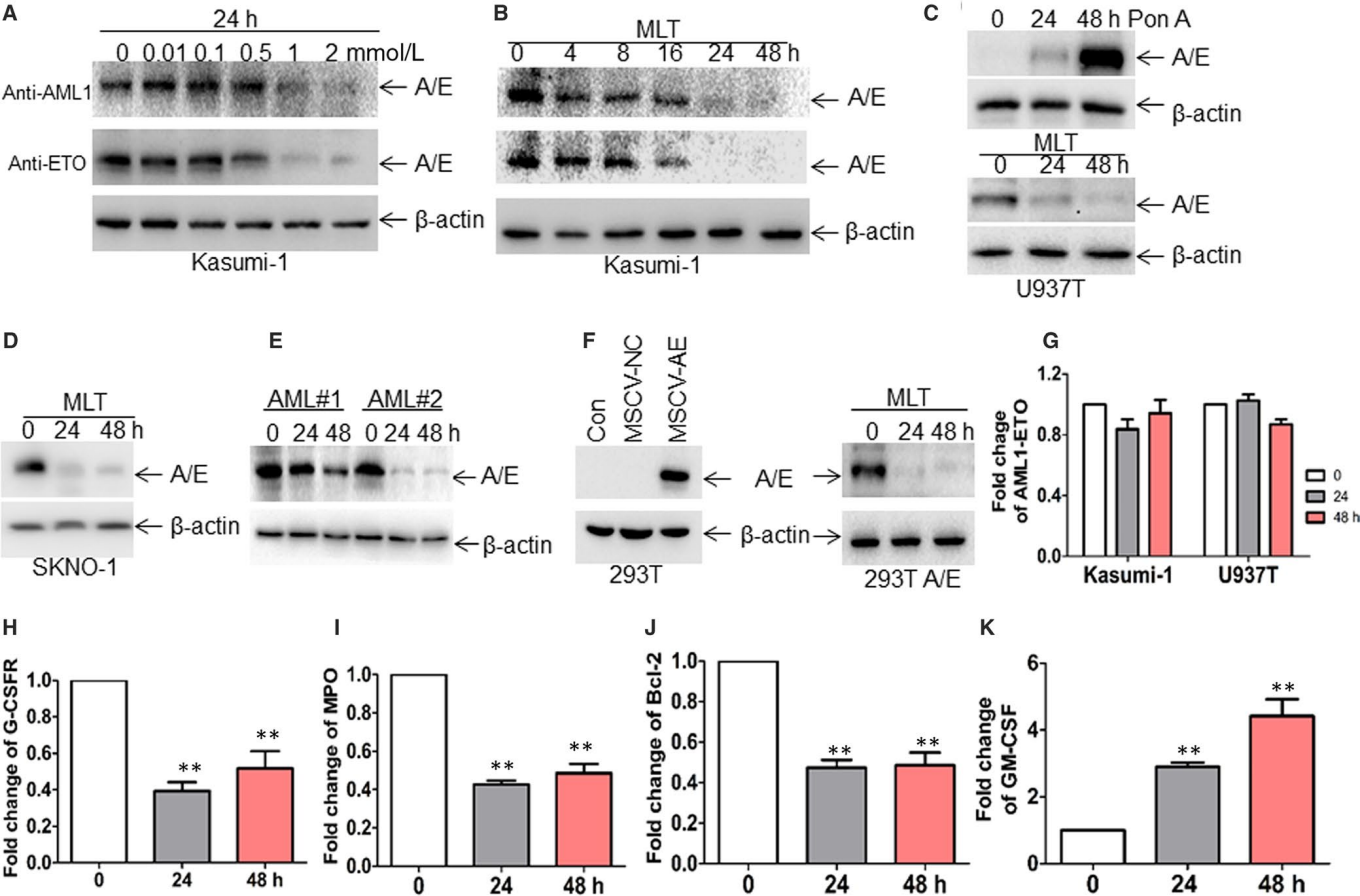
Melatonin (MLT) rapidly induces the degradation of AML1‐ETO protein. (A,B), The expression of AML1‐ETO fusion protein (A/E) was measured by western blot through anti‐AML1 and anti‐ETO antibody in Kasumi‐1 cells treated with different concentrations of MLT for 24 h (A) and with 1 mmol/L MLT for different times (B), respectively. The following blots for AML1‐ETO were performed through anti‐AML1 antibody. (C), The protein level of AML1‐ETO was detected in U937T cells treated with 5 μmol/L Pon A for 24 and 48 h (upper blots). The expression of AML1‐ETO was detected in Pon A‐treated U937T cells, which were incubated with 1 mmol/L MLT for 24 and 48 h (down blots). (D), The expression of AML1‐ETO was detected in SKNO‐1 cells, which were incubated with 1 mmol/L MLT for 24 and 48 h (E) Bone marrow mononuclear cells (blasts >70%) were isolated from two AML patients with positive AML1‐ETO. The protein expression of AML1‐ETO was measured in the two AML blasts treated with 1 mmol/L MLT for 24 and 48 h. (F) 293T cells were transduced with MSCV‐GFP‐A/E or negative control, followed by detection of AML1‐ETO expression (left blots). 293T cells expressing AML1‐ETO (293T A/E) were treated with 1 mmol/L MLT for 24 and 48 h, and then AML1‐ETO expression was measured (Right blots). (G) The transcriptional expression of *AML1‐ETO* was measured in Kasumi‐1 and U937T cells treated with 1 mmol/L MLT for 24 and 48 h by Quantitative real‐time PCR (qRT‐PCR). (H‐K), The mRNA expressions of granulocyte colony‐stimulating factor receptor (*G‐CSFR*), myeloperoxidase (*MPO*), *Bcl‐2* and granulocyte‐macrophage colony‐stimulating factor (*GM‐CSF*) were detected by qRT‐PCR in Kasumi‐1 cells treated with 1 mmol/L MLT for 24 and 48 h. **P < 0.01 vs untreated cells

To determine whether AML1‐ETO protein was degraded by MLT, AML1‐ETO protein expression was analysed in Kasumi‐1 cells carrying endogenous AML1‐ETO, which were treated with 0.01, 0.1, 0.5, 1, 2 mmol/L MLT for 24 hours. As indicated in Figure 1A, MLT (0.5 mmol/L) started to degrade AML1‐ETO protein and MLT (1 and 2 mmol/L) almost completely degraded AML1‐ETO protein. Furthermore, MLT (1 mmol/L) degraded AML1‐ETO protein at 4 hours and almost completely degraded AML1‐ETO protein at 24 and 48 hours (Figure 1B). Thus, this concentration of MLT (1 mmol/L) was used for the next tests. To address whether MLT could degrade exogenous AML1‐ETO protein, U937‐AML1‐ETO stable transformants (U937‐A/E9/14/18, U937T) were treated with MLT because U937T conditionally expresses the AML1‐ETO protein after treated by Pon A.^17^ As expected, Pon A effectively induced AML1‐ETO expression (Upper blots, Figure 1C). Accordingly, MLT degraded AML1‐ETO protein in Pon A‐induced U937T cells (Down blots, Figure 1C). To further determine the degradation of AML1‐ETO protein by MLT, SKNO‐1 cell line bearing AML1‐ETO was used. MLT (1 mmol/L) also degraded AML1‐ETO protein expression at 24 and 48 hours in SKNO‐1 cells (Figure 1D).

To further confirm whether MLT degraded AML1‐ETO protein in primary leukemic samples, BM leukaemia blasts from two AML patients carrying AML1‐ETO were treated with MLT for 24 and 48 hours. Similarly, MLT induced the degradation of AML1‐ETO protein in primary AML blasts (Figure 1E). We then asked whether MLT degraded AML1‐ETO protein in non‐leukemic cells. 293T cells were transduced with MSCV‐GFP‐AML1‐ETO to continuously express AML1‐ETO. Western blot indicated successful expression of AML1‐ETO in 293T cells (Left blots, Figure 1F). MLT also degraded AML1‐ETO protein in 293T‐A/E cells (Right blots, Figure 1F). Finally, *AML1‐ETO* transcriptional level was detected in MLT‐treated leukemic cells. However, MLT slightly down‐regulated *AML1‐ETO* mRNA expression in Kasumi‐1 and U937T cells (Figure 1G).

AML1‐ETO contributes to the proliferation and the self‐renewal through modulating different target genes. For example, AML1‐ETO induces the expression of *Bcl‐2*, granulocyte colony‐stimulating factor receptor (*G‐CSFR*), and *MPO* and inhibits the transactivation of the granulocyte‐macrophage colony‐stimulating factor (*GM‐CSF*).^23^ Next, we determined whether AML1‐ETO‐targeted genes were regulated by MLT. As expected, MLT decreased the mRNA expressions of *G‐CSFR*, *MPO*, and *Bcl‐2* (Figure 1H‐J). Meanwhile, MLT increased the expression of *GM‐CSF* in Kasumi‐1 and U937T cells (Figure 1K).

### 3.2 Anti‐leukaemia activity by MLT

**Figure 2:**
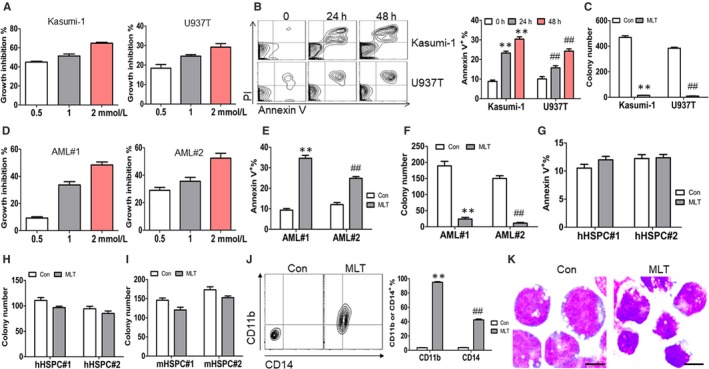
Anti‐leukaemia activity of Melatonin (MLT). (A), Cell growth was measured by CCK‐8 in Kasumi‐1 and U937T cells treated with 0.5, 1, 2 mmol/L MLT for 24 h. (B), Apoptosis was measured by Annexin V/PI staining in Kasumi‐1 and U937T cells treated with or without 1 mmol/L MLT for 24 and 48 h. Shown is the representative plots (Left) and the summary of Annexin V^+^ cells (Right). **and ^##^ *P* < 0.01 vs untreated cells, respectively. (C), Kasumi‐1 and U937T cells (2 × 10^3^) were plated on methylcellulose medium treated with or without 1 mmol/L MLT and colonies were counted. ** and ^##^ *P* < 0.01 vs untreated cells, respectively. (D), Cell growth was measured by CCK‐8 in two primary AML blasts with positive AML1‐ETO, which were treated with or without 0.5, 1, 2 mmol/L MLT for 24 h. (E), Apoptosis was measured by Annexin V/PI staining in two primary AML blasts bearing AML1‐ETO treated with or without 1 mmol/L MLT for 24 h. ** and ^##^ *P* < 0.01 vs untreated cells, respectively. (F), CD34^+^ cells (1 × 10^3^) were isolated from two primary AML blasts bearing AML1‐ETO and were plated on methylcellulose medium incubated with or without 1 mmol/L MLT for colony counting. ** and ^##^ *P* < 0.01 vs untreated cells, respectively. (G), Apoptosis was measured by Annexin V/PI staining in two CD34^+^ cells, which were isolated from two umbilical cord blood, treated with or without 1 mmol/L MLT for 24 h. (H), CD34^+^ cells (1 × 10^3^) were isolated from two cord blood and were plated on methylcellulose medium treated with or without 1 mmol/L MLT for colony counting. (I), Lineage^−^ cells (Lin^−^) were isolated from BM mononuclear cells of two wild‐type C57 mice. Lin^−^ cells (2 × 10^3^) were plated on methylcellulose medium for colony formation treated with or without 1 mmol/L MLT, followed by colony counting. (J), CD11b and CD14 were detected in Kasumi‐1 cells treated with or without 1 mmol/L MLT for three days. Shown is the representative plot (Left) and the summary of CD11b^+^ and CD14^+^ cells (Right). ** and ^##^ *P* < 0.01 vs untreated cells, respectively. (K), Wright‐Giemsa staining for Kasumi‐1 cells treated with or without 1 mmol/L MLT for three days. Scale bars represent 10 μmol/L

To determine whether MLT has potential anti‐leukaemia activity in leukemic cells bearing AML1‐ETO, apoptosis, proliferation and colony formation were analysed in MLT‐treated leukemic cell lines and primary AML blasts. MLT moderately inhibited cell growth in Kasumi‐1 and U937T cells in a concentration‐dependent manner (Figure 2A). Similarly, MLT moderately induced apoptosis in Kasumi‐1 and U937T cells (Figure 2B). Furthermore, colony formation was measured in MLT‐treated leukaemia cells. Interestingly, MLT almost completely inhibited the colony formation in Kasumi‐1 and U937T (Figure 2C). To further explore the anti‐leukaemia activity of MLT, primary AML blasts bearing AML1‐ETO were treated with MLT. Also, MLT decreased proliferation (Figure 2D), induced apoptosis (Figure 2E) and substantially reduced the colony formation (Figure 2F) in two primary blasts from AML patients with AML‐ETO.

To assess whether MLT affects the apoptosis and colony formation in normal HSPCs, apoptosis and colony formation were analysed in MLT‐treated CD34^+^ cells from human cord blood as normal HSPCs. Treatment of MLT slightly induced apoptosis (Figure 2G) and decreased colony formation almost 10%‐20% in two normal human CD34^+^ HSPCs (Figure 2H). We further assessed the effects of MLT on mouse lineage^−^ cells (Lin^−^) as normal HSPCs and found that MLT exhibited minimal effects on the colony formation in two normal mouse HSPCs (Figure 2I).

Because siRNA‐mediated suppression of AML1‐ETO enhances differentiation,^24^ we hypothesized that MLT induces the differentiation in leukaemia cells. For this purpose, cell surface markers and cell morphology were analysed in MLT‐treated leukaemia cells. MLT treatment significantly increased the expressions of CD11b and CD14 (Figure 2J), two markers for myeloid maturation,^25^ in Kasumi‐1 cells for three days. Meanwhile, cellular morphology was evaluated under light microscopy. MLT induced nuclear condensation and decreased cell size (Figure 2K), suggesting that MLT induces cell differentiation.

### 3.3 MLT‐induced inhibition of AML1‐ETO is independent of caspase‐3 activation, ubiquitin‐proteasome system and MT1/2 receptor

**Figure 3:**
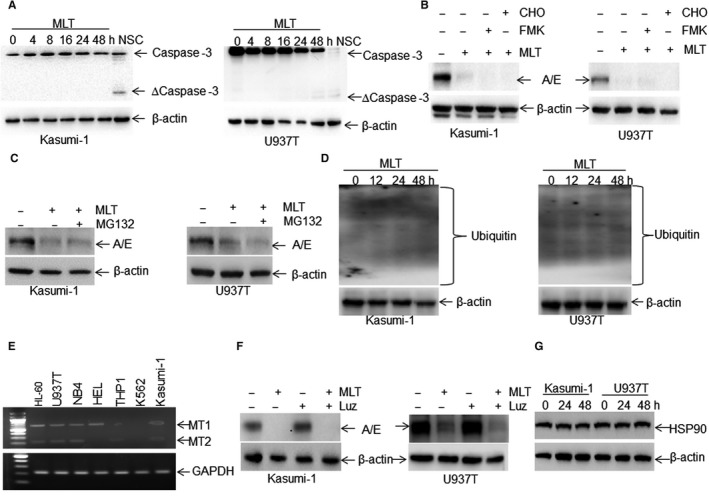
Melatonin (MLT)‐induced degradation of AML1‐ETO is independent of caspase‐3 activation, ubiquitin‐proteasome system and MT1/2 receptor. (A), Total caspase‐3 and active caspase‐3 were detected in Kasumi‐1 and U937T cells incubated with 1 mmol/L MLT for different times. NSC606985‐treated cell lysates from Kasumi‐1 and U937T were used for positive controls. (B), AML1‐ETO expression was detected in Kasumi‐1 and U937T cells, which were pre‐treated with pan‐caspase inhibitor Z‐VAD‐FMK (20 μmol/L) and caspase‐3 inhibitor Ac‐DEVD‐CHO (20 μmol/L) for 1 h, followed by MLT (1 mmol/L) treatment for 24 h. (C), The protein expression of AML1‐ETO was measured in Kasumi‐1 and U937T cells, which were treated with 1 mmol/L MLT for 24 h. The proteasome inhibitor MG132 (5 μmol/L) was added or not 4 h before cell harvest. (D), The ubiquitinated proteins were measured in leukemic cells treated with MLT or not for 12, 24 and 48 h. (E) The transcriptional expressions of *MT1* and *MT2* were measured by RT‐PCR in several leukaemia cell lines. (F), The protein expression of AML1‐ETO was detected in Kasumi‐1 and U937T cells treated with 1 mmol/L MLT, MT1/2 antagonist luzindole (Luz, 5 μmol/L) and MLT+Luz for 24 h. (G), heat shock protein 90 (HSP90) protein expression was measured in Kasumi‐1 and U937T cells treated with or without 1 mmol/L MLT for 24 and 48 h

As documented,^17^ AML1‐ETO fusion protein can be cleaved to four fragments by activated caspase‐3 during apoptosis induction. To exclude the possibility that AML1‐ETO protein was also degraded by activated caspase‐3 during MLT‐induced apoptosis, the expression of activated caspase‐3 was detected in MLT‐induced leukaemia cells. Different treatment times with MLT had no significant effect on caspase‐3 activation in Kasumi‐1 cells, but slightly induced the activated caspase‐3 in U937T cells at 48 hours (Figure 3A). NSC606985, a camptothecin ester derivative, was used as a positive control of activated caspase‐3.^26^ To further confirm that MLT‐induced degradation of AML1‐ETO is independent of activated caspase‐3, Z‐VAD‐FMK (FMK), a specific pan‐caspase inhibitor, and Ac‐DEVD‐CHO (CHO), a specific caspase‐3 inhibitor, were used to inhibit MLT‐induced apoptosis. FMK and CHO did not rescue MLT‐induced degradation of AML1‐ETO in Kasumi‐1 and U937T cells (Figure 3B), suggesting that MLT‐induced degradation of AML1‐ETO is independent of activated caspase‐3.

The observation that MLT mainly decreased the protein expression of AML1‐ETO but only slightly decreased its mRNA expression prompted us to determine whether MLT induces the degradation of AML1‐ETO protein at the post‐transcriptional level, which is mainly mediated by the ubiquitin‐proteasome system,^27^ a major system for the degradation of proteins. For this purpose, Kasumi‐1 and U937T cells were treated with MLT in the presence or absence of MG132, a prototypical proteasome inhibitor.^28^ However, MG132 did not block MLT‐induced degradation of AML1‐ETO protein (Figure 3C). To further determine whether MLT enhances the degradation rate, the ubiquitinated proteins were measured in leukaemia cells treated with or without MLT for 12, 24 and 48 hours. MLT did not substantially enhance the ubiquitinated proteins (Figure 3D).

As reported,^14^ MLT affects the physiological function mainly through interaction with two membrane receptors, MT1 and MT2. Thus, we further investigated whether MLT‐induced degradation of AML1‐ETO depends on MT1 and MT2. We firstly detected the mRNA expressions of *MT1* and *MT2* in multiple types of leukaemia cell lines. The transcriptional expressions of *MT1* and *MT2* were easily detectable in most leukaemia cell lines including Kasumi‐1 and U937T cells but not in K562 cells by RT‐PCR (Figure 3E). However, luzindole, a specific MT1 and MT2 antagonist,^29^ did not prevent MLT‐induced degradation of AML1‐ETO in Kasumi‐1 and U937T cells (Figure 3F).

As documented,^30^ AML1‐ETO interacts with heat shock protein 90 (HSP90), an important molecular chaperone that plays a key role in the conformational maturation and stabilization of signalling proteins. Furthermore, 17‐allylamino‐geldanamycin (17‐AAG), a HSP90 antagonist, induces the degradation of AML1‐ETO.^30^ To further exclude the possibility that MLT degraded the AML1‐ETO protein through degrading HSP90, the expression of HSP90 was measured in Kasumi‐1 and U937T cells treated with MLT for 24 and 48 hours. However, MLT failed to affect the protein expression of HSP90 (Figure 3G).

### 3.4 MLT degrades AML1‐ETO through up‐regulation of miR‐193a

**Figure 4:**
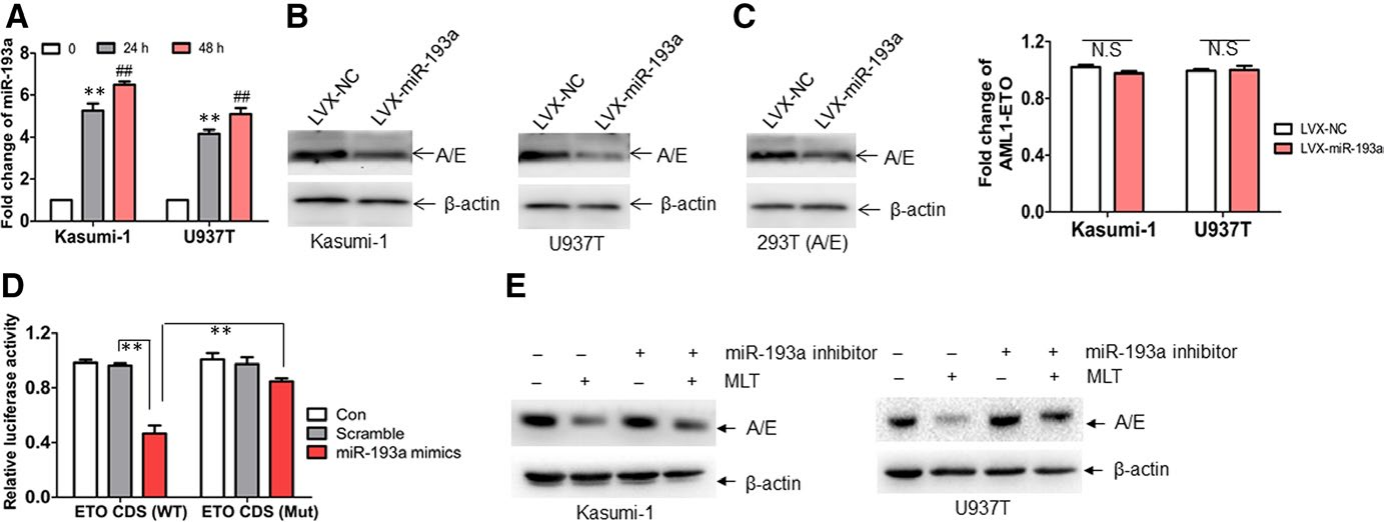
miR‐193a mediates the inhibition of AML1‐ETO protein by Melatonin (MLT). (A), The expression of miR‐193a was detected in Kasumi‐1 and U937T cells treated with or without 1 mmol/L MLT for 24 and 48 h. ** and ^##^ *P* < 0.01 vs untreated cells, respectively. (B), Kasumi‐1 and U937T cells were transduced with lentivirus vector LVX‐miR‐193a expressing miR‐193a or negative control LVX‐NC. The protein expression of AML1‐ETO was measured in transduced leukaemia cells. (C), The protein level of AML1‐ETO was detected in 293T A/E cells, which were transduced with LVX‐miR‐193a or NC. (D), 293T cells were transfected with psiCHECK‐ETOCDS (WT) or psiCHECK‐ETOCDS (Mut) for 24 h, followed by the transfection with miR‐193a mimic or Scramble for another 24 h. Firefly and Renilla luciferase activities were both detected and histograms showed that the Firefly luciferase activities were normalized to Renilla luciferase activities. ** and ^##^ *P* < 0.01. (E), Kasumi‐1 and U937T cells were transfected with a specific inhibitor of miR‐193a and then treated with or without 1 mmol/L MLT for 24 h, followed by detection of AML1‐ETO protein expression

Our data indicated that MLT‐induced degradation of AML1‐ETO is independent of activated caspase‐3, ubiquitin‐proteasome system and MT1/2 receptors. These results prompted us to explore whether MLT induced the degradation of AML1‐ETO through up‐regulation of miRNAs, which bind 3'‐UTR or CDS of target gene mRNA, resulting in translational repression or mRNA degradation.^31^ Because miR‐193a (miR‐193a‐3p) has been reported to repress the expression of AML1‐ETO through binding 3'‐UTR of *ETO*,^32^ we hypothesized that MLT induced the degradation of AML1‐ETO via up‐regulation of miR‐193a. For this purpose, miR‐193a expression was measured in MLT‐treated leukaemia cells. MLT increased the expression of miR‐193a about four**‐**sixfolds (Figure 4A). To confirm that miR‐193a inhibits AML1‐ETO expression, Kasumi‐1 and U937T cells were transduced with lentivirus vector LVX‐miR‐193a, which overexpresses miR‐193a, or negative control LVX‐NC (NC). Overexpression of miR‐193a down‐regulated the expression of AML1‐ETO in Kasumi‐1 (Left blots, Figure 4B) and U937T cells (Right blots, Figure 4B), as well as in 293T (A/E) cells (Figure 4C).

MiR‐193a represses the expression of AML1‐ETO through binding 3'‐UTR of *ETO* in Kasumi‐1 cells expressing endogenous AML1‐ETO.^32^ However, miR‐193a inhibited AML1‐ETO protein expression in U937T (Right blots, Figure 4B) and 293T (A/E) (Figure 4C) cells, which carry CDS of *ETO* but not 3'‐UTR of *ETO*. Thus, we asked whether miR‐193a binds CDS of *ETO*. To investigate this, we searched the possible binding sites through miRwalk (http://mirwalk.umm.uni-heidelberg.de/) and found the putative binding sites on CDS of *ETO* (Figure S1A). To further confirm whether miR‐193a binds CDS of *ETO*, CDS including putative miR‐193a‐binding sites was subcloned into psiCHECK vector to produce psiCHECK‐ETO (CDS), which was co‐transfected together with miR‐193a or Scramble into 293T cells. miR‐193a decreased luciferase activities about 60% (Figure 4D). However, the decreased luciferase activity was almost abolished by the mutation of putative miR‐193a‐binding sites (Figure 4D).

To further confirm that miR‐193a plays an important role in MLT‐induced degradation of AML1‐ETO, a specific inhibitor of miR‐193a was transfected in MLT‐treated cells and AML1‐ETO was measured. Inhibition of miR‐193a partially prevented MLT‐induced degradation of AML1‐ETO in Kasumi‐1 (Left blots, Figure 4E) and U937T cells (Right blots, Figure 4E).

### 3.5 Effects of MLT in Kasumi‐1 and U937T‐GFP xenograft mouse model

**Figure 5:**
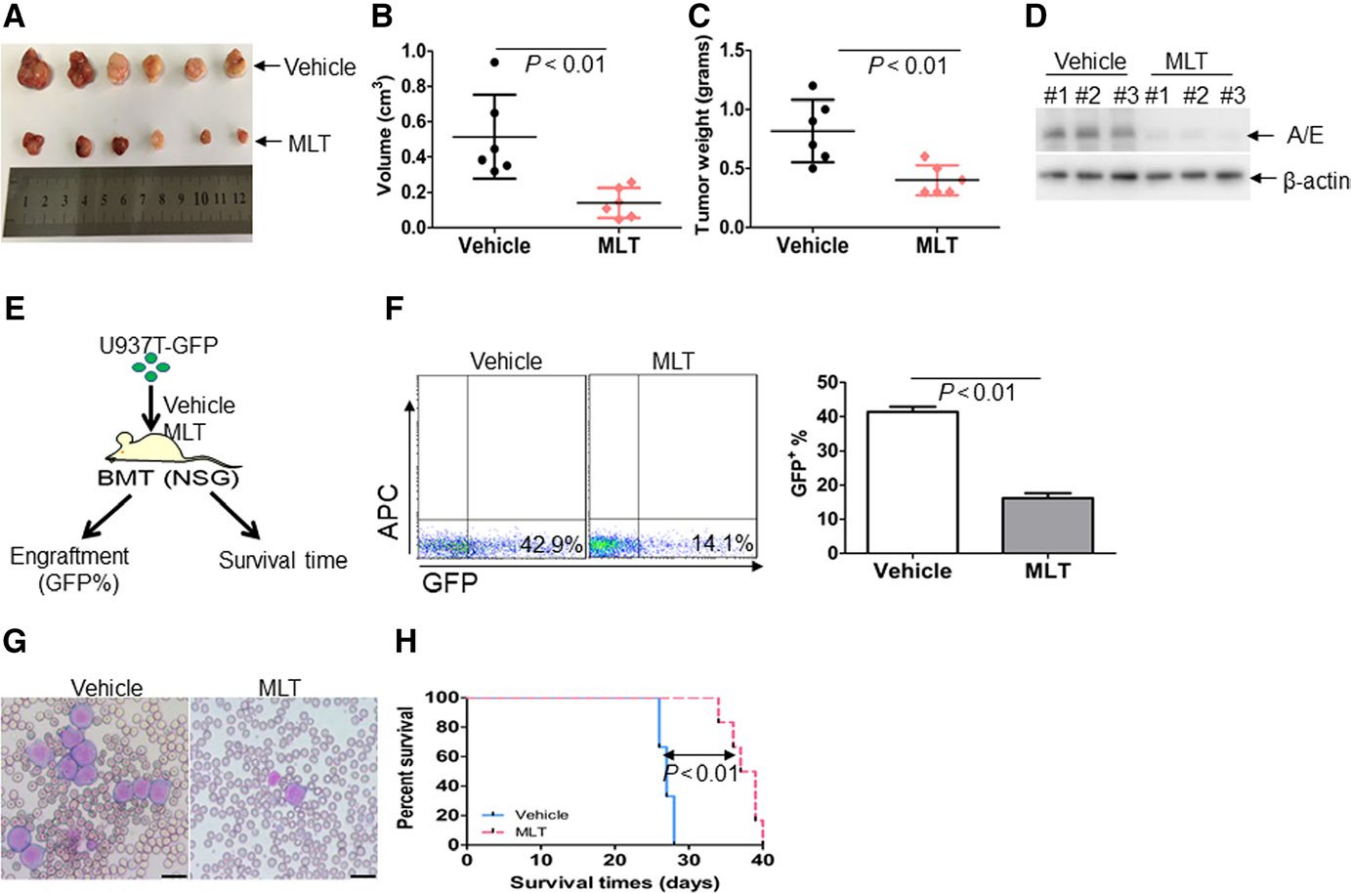
The anti‐leukaemia effects of Melatonin (MLT) in Kasumi‐1‐ and U937T‐GFP‐inoculated mice. (A), A photograph of tumours in MLT‐treated mice and vehicle mice. (B), Volumes of all tumours were measured when the experiment was terminated after tumour cell inoculation. (C), Net weights of all tumours were measured at the termination of the experiment. (D), The protein levels of AML1‐ETO were detected in tumour lysates from MLT‐treated mice and vehicle mice. (E), NSG mice were divided into two groups as vehicle or MLT‐treated mice after inoculation with 1 × 10^6^ U937T‐GFP cells. (F), GFP^+^ cells were measured in peripheral blood from vehicle and MLT‐treated mice. Shown is the representative plot (Left) and summary of GFP^+^ cells (Right). (G) Blood cells from vehicle and MLT‐treated mice were stained by Wright's staining. Scale bars represent 10 μmol/L. (H), The overall survival time in vehicle and MLT‐treated mice

To explore whether MLT could reduce the tumourigenicity in vivo, Kasumi‐1 cells were injected subcutaneously into the right flanks of mice to produce xenograft mouse model followed by MLT treatment or not. MLT treatment substantially reduced average tumour volume by 45.7% compared with vehicle mice (Figure 5A,B). Also, MLT treatment led to a 41.2% decrease in average tumour weight (Figure 5C). Because MLT decreases the expression of AML1‐ETO in vitro, we then determined whether MLT reduced the AML1‐ETO expression in vivo. As expected, the protein levels of AML1‐ETO were significantly decreased in the tumours from MLT‐treated mice compared with vehicle mice (Figure 5D). These results indicate that MLT inhibits the tumour growth of Kasumi‐1 cells in vivo.

To further explore the anti‐leukaemia activity, U937 cells were transduced with MSCV‐GFP‐IRES‐AML1‐ETO to produce U937T‐GFP cells, which continuously expresses both GFP (Figure S2A) and AML1‐ETO (Figure S2B). U937T‐GFP cells were transplanted into NSG mice and then the mice were treated with or without MLT (Figure 5E). GFP^+^ cells were detected to reflect the infiltration of human leukaemia blasts. MLT decreased leukaemia cells in NSG mice by about 60% (Figure 5F). Furthermore, MLT substantially weakened the expansion of engrafted leukemic cells in peripheral blood (Figure 5G) and prolonged the overall survival (Figure 5H).

### 3.6 MLT decreases leukemic blasts, induces differentiation and prolongs survival in AML1‐ETO‐induced murine leukaemia

**Figure 6:**
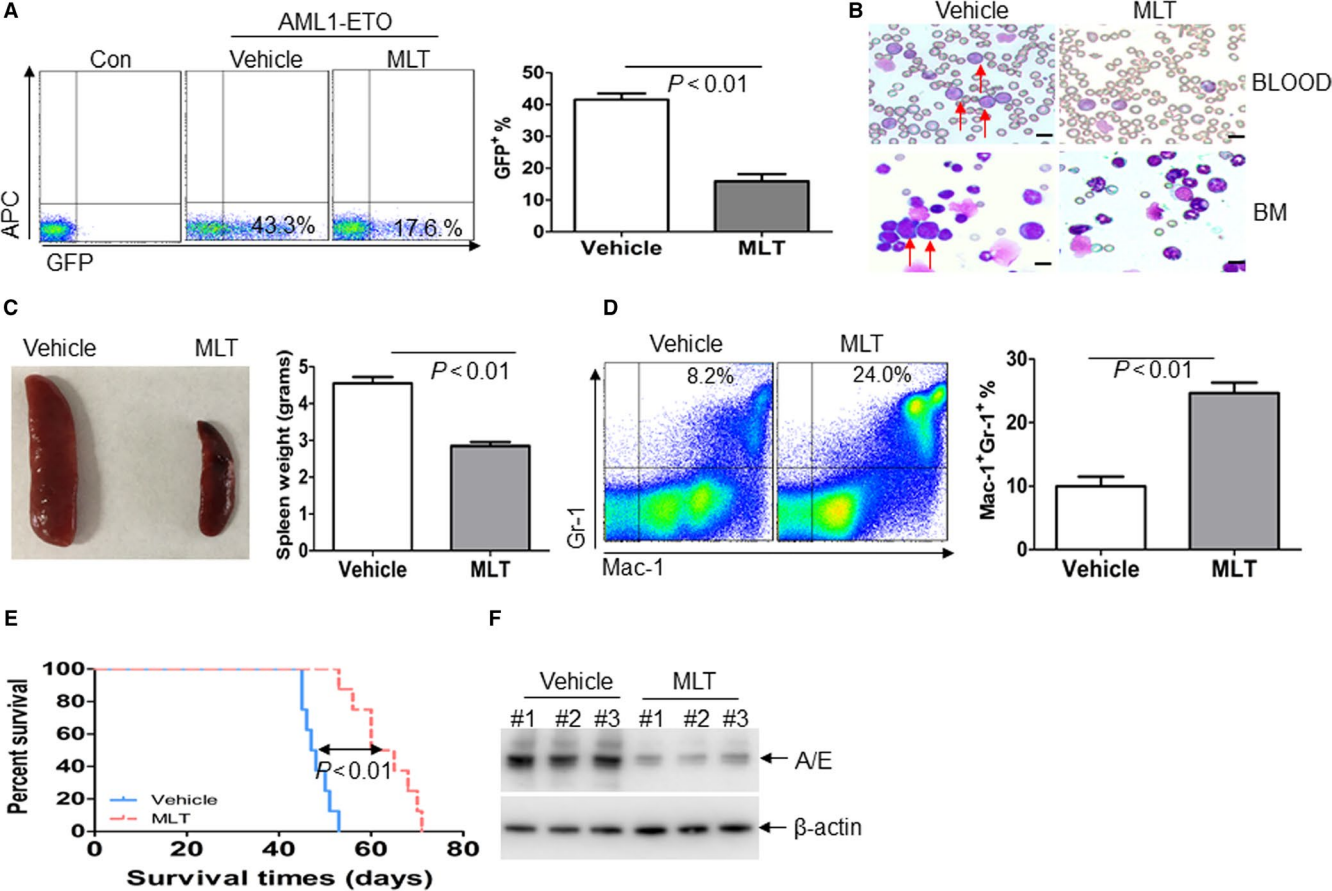
Melatonin (MLT) decreases leukemic blasts, induces differentiation, and prolongs survival in AML1‐ETO‐induced murine leukaemia. (A), GFP^+^ cells were measured in BM cells from AML1‐ETO‐expressing mice leukaemia treated with or without MLT when vehicle mice developed full‐blown leukaemia (N = 4 for each group). Shown is the representative plot (Left) and summary of GFP^+^ cells (Right). (B), Wright‐Giemsa staining of blood smear and bone marrow smear in AML1‐ETO‐expressing mice leukaemia treated with or without MLT. Scale bars represent 10 μmol/L. (C), A representative image of spleen (Left) and summary of spleen weight (Right) from AML1‐ETO‐expressing murine leukaemia treated with or without MLT (N = 4 for each group). (D), Percentage of Gr‐1^+^Mac‐1^+^ cells were detected by flow cytometry in BM blasts from vehicle or MLT‐treated leukaemia mice (N = 4 for each group). Shown is the representative plot (Left) and summary of Gr‐1^+^Mac‐1^+^ cells (Right). (E), Overall survival was analysed in the vehicle and MLT‐treated murine leukaemia (N = 8 for each group). (F), The protein expression of AML1‐ETO was detected in BM blasts from vehicle and MLT‐treated mice

Based on the observation that MLT almost completely inhibits colony formation in leukaemia cells, we speculate that MLT presents anti‐leukaemia activity via suppressing self‐renewal of leukaemia stem cells or leukaemia progenitor cells. As reported, AML1‐ETO can efficiently induce mice leukaemia in cooperation with *WT1* gene (Wilm's tumour‐1).^5^ To construct AML1‐ETO‐expressing mice leukaemia model, Lin^−^ cells were isolated and transduced with AML1‐ETO plus WT1 and xenografted in recipient mice (Figure S3A). After the mice developed full‐blown leukaemia, GFP^+^ cells expressing AML1‐ETO were isolated and intravenously injected into lethally irradiated secondary recipient mice followed by MLT treatment or not (Figure S3A). Western blots for AML1‐ETO and WT1 in BM cells from leukaemia mice and normal mice demonstrate the successful transfection of AML1‐ETO and WT1 (Figure S3B). GFP^+^ cells were measured in bone marrow when vehicle mice developed full‐blown leukaemia. MLT markedly reduced the percentage of GFP^+^ cells, which represent immature leukaemia cells (Figure 6A). Furthermore, MLT substantially reduced the infiltration of leukaemia blasts, which have abnormal nuclei and cytoplasmic vacuoles, in peripheral blood (Figure 6B). Also, MLT suppressed immature blasts in bone marrow (Figure 6B). Massive leukemic infiltrations were closely packed in dense sheets and completely destroyed the normal architecture of the spleen in vehicle mice (Figure S3C). MLT inhibited the leukemic infiltration in spleen (Figure S3C) and markedly reduced spleen weight (Figure 6C). To determine whether MLT induced differentiation of mice leukaemia cells, Mac‐1^+^Gr‐1^+^ cells, which represent differentiated myeloid cells, was measured in bone marrow cells. MLT increased the percentage of Mac‐1^+^Gr‐1^+^ cells about threefold (Figure 6D). To determine whether MLT extends the survival time of leukaemia mice, overall survival was analysed. MLT significantly extended the overall survival of mice leukaemia. Median survival of MLT vs vehicle was 62.5 days vs 47.5 days, respectively (*P* < 0.01, Figure 6E). Finally, the protein expression of AML1‐ETO was measured in MLT‐treated mice and vehicle mice. As expected, MLT decreased the protein expression of AML1‐ETO (Figure 6F).

### 3.7 MLT decreases the long‐term self‐renewal of LSC in AML1‐ETO‐induced murine leukaemia

**Figure 7:**
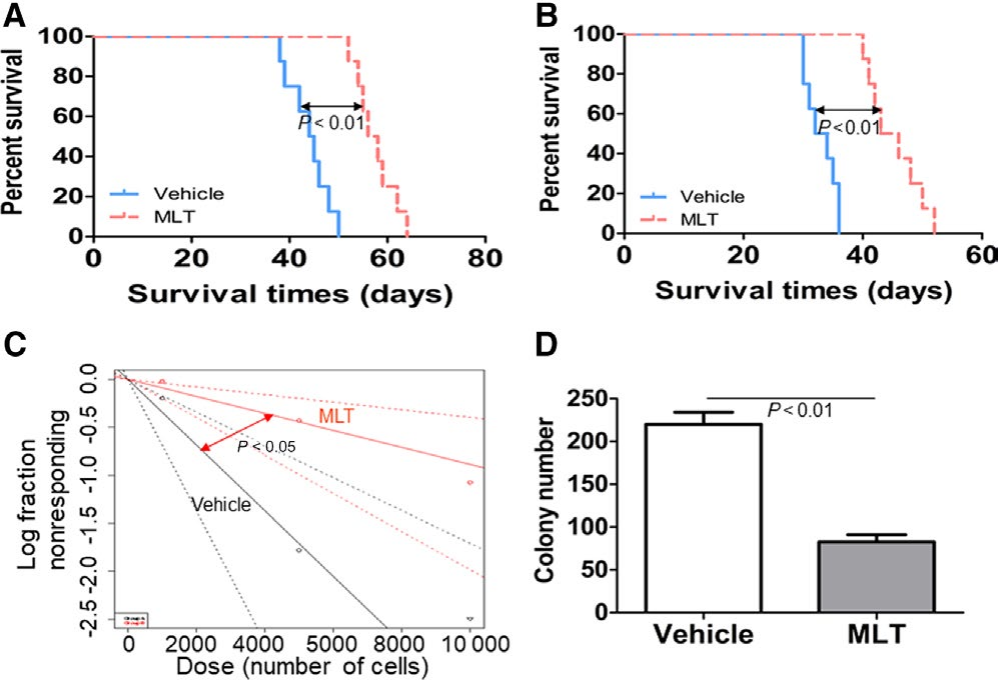
Melatonin (MLT) decreases the long‐term self‐renewal of leukaemia stem cell (LSC) in AML1‐ETO‐induced murine leukaemia. (A), Overall survival was analysed in the secondary BMT (N = 8 for each group). (B), Overall survival was analysed in the tertiary BMT (N = 8 for each group). (C), Limiting dilution assay of BM blasts from vehicle or MLT‐treated murine leukaemia. LSC frequencies and the *P*‐value were calculated by L‐calc software. (D) Colony formation was counted in GFP^+^ cells (1 × 10^3^) from vehicle or MLT‐treated murine leukaemia (N = 4 for each group).

To assess the impact of MLT on long‐term self‐renewal of LSC with AML1‐ETO, we performed serial mouse BMT assays. In the secondary mouse BMT assay, vehicle or MLT‐treated BM blasts isolated from primary leukaemia mice were transplanted to lethally irradiated wild‐type recipient mice. Vehicle and MLT‐treated mice developed leukaemia much faster than the corresponding primary BMT groups, respectively (Figure 7A vs Figure 6E). Furthermore, MLT treatment significantly prolonged the overall survival time compared with vehicle mice (Figure 7A, median survival time of MLT vs vehicle was 57 days vs 44.5 days, respectively; *P* < 0.01). Then, tertiary mouse BMT was performed using secondary leukaemia BM blasts as donor cells. Vehicle and MLT‐treated mice developed AML much faster compared with the secondary BMT groups (Figure 7B vs Figure 6E). Most importantly, MLT substantially prolonged the overall survival time compared with vehicle mice (Figure 7B, median survival of MLT vs vehicle was 47 days vs 34.5 days, respectively; *P* < 0.01).

To directly compare the effects of MLT treatment on the frequency of LSC, we conducted limiting dilution assays with mouse BM leukemic cells collected from tertiary BMT recipients as donor cells. Transplantation results showed that LSC frequency was decreased in MLT‐treated mice vs vehicle mice (1/11287 vs 1/2924, *P* < 0.05, Figure 7C). Finally, to investigate the functional role of MLT on LSC function, we evaluated the colony formation ability of MLT‐treated mice and vehicle mice. GFP^+^ cells from MLT‐treated mice formed colonies at lower frequency than those from vehicle mice (Figure 7D).

### 3.8 MLT inhibits self‐renewal by reducing β‐catenin activity

As documented,^9^ β‐catenin is essential for the hematopoietic self‐renewal of leukaemia cells with AML1‐ETO. To investigate whether MLT reduces β‐catenin activity, the protein expression of β‐catenin was detected in Kasumi‐1 and U937T cells treated with MLT. MLT decreased the protein expression of β‐catenin in Kasumi‐1 and U937T cells (Figure S4A). To further investigate the role of β‐catenin on the anti‐self‐renewal by MLT, Kasumi‐1 and U937T cells were transduced with LVX‐NC or LVX‐GFP‐β‐catenin. Western blot indicated successful overexpression of β‐catenin (Figure S4B). Importantly, overexpression of β‐catenin partially prevented MLT‐induced inhibition of colony formation (Figure S4C,D).

β‐catenin was decreased as a consequence of the degradation of AML1‐ETO.^9^ To determine whether MLT directly regulates the expression of β‐catenin independent of degradation of AML1‐ETO in leukemic cells, K562, THP1, and U937 cells, which have not express AML1‐ETO, were incubated with MLT. MLT treatment slightly down‐regulated the expression of β‐catenin in these leukemic cells (Figure S5A). Therefore, we speculate that MLT decreases the expression of β‐catenin mainly through the degradation of AML1‐ETO.

## 4 DISCUSSION

AML1‐ETO is an important factor for the leukemogenesis through enhancing self‐renewal of LSC in AML patients with t (8;21). Inhibiting AML1‐ETO may provide a potential therapy for these AML patients. Here, we report that MLT strongly decreases endogenous and exogenous expressions of AML1‐ETO protein in leukaemia cell lines and primary AML blasts. MLT decreases AML1‐ETO through up‐regulation of miR‐193a, which inhibits AML1‐ETO expression via binding its CDS and 3'UTR sites. In addition, MLT decreases the expression of β‐catenin, which has been reported as downstream of AML1‐ETO,^9^ suggesting that MLT presents anti‐self‐renewal of LSC through miR‐193a‐AML1‐ETO‐β‐catenin axis in AML1‐ETO‐expressing cells (Figure S4E). Therefore, MLT might provide the lead natural compound for the development of AML1‐ETO‐targeted agents.

miRNAs have recently been found to play an important role in biological regulations such as apoptosis, proliferation, and differentiation in haematological cells by modulating the expression of oncogenes or tumour suppressors.^21^, ^33^ We identified miR‐193a as a candidate miRNA regulated by MLT. AML1‐ETO triggers the silencing of miR‐193a and in contrast, miR‐193a decreases AML1‐ETO expression via targeting its binding sites, suggesting that a feedback circuitry is formed in AML cells bearing AML1‐ETO.^32^ It is possible that MLT‐induced degradation of AML1‐ETO disrupts the feedback circuitry and increases the expression of miR‐193a. However, inhibition of miR‐193a partially blocks MLT‐induced degradation of AML1‐ETO protein in leukaemia cells, suggesting that MLT inhibits AML1‐ETO protein partially through up‐regulation of miR‐193a. Thus, we speculate that firstly, MLT increases the expression of miR‐193a, which reduces the protein expression of AML1‐ETO. Secondly, the feedback circuitry between AML1‐ETO and miR‐193a is disrupted, resulting in the further down‐regulation of AML1‐ETO protein. However, further studies are still required to determine whether other miRNAs besides miR‐193a mediate MLT‐induced inhibition of AML1‐ETO protein.

MLT has been reported to present anti‐leukaemia activity through different mechanisms.^15^, ^34^, ^35^ However, whether MLT inhibits self‐renewal of LSC is not determined. The most important finding of this study is that MLT suppresses colony formation, decreases frequency of LSC, and extends overall survival in AML1‐ETO‐induced murine leukaemia. AML1‐ETO enhances hematopoietic self‐renewal and leukemogenesis through β‐catenin signaling pathway.^9^ Our results indicate that MLT decreases the expression of β‐catenin and overexpression of β‐catenin partially prevents MLT‐induced anti‐self‐renewal, suggesting that MLT presents anti‐self‐renewal activity through AML1‐ETO‐β‐catenin axis. Consistent with our results, MLT has been reported to promote the anticancer effect of paclitaxcel in brain cancer stem cells.^36^ Most importantly, MLT has little effect on the colony formation in normal human and murine HSPC. Thus, MLT is considered as a low‐toxicity protective agent against leukaemia cells. For example, MLT ameliorates the toxicity induced by therapeutic drugs^37^ and X‐ray radiation.^34^ Therefore, MLT should be a potential anti‐LSC agent in AML with t (8;21) translocation without severe side‐effects.

Targeting AML1‐ETO has achieved potential beneficial effector for AML patients with AML1‐ETO. For example, oridonin shows significant anti‐leukaemia ability in vitro and in vivo through inducing the degradation of AML1‐ETO oncoprotein.^23^ Depsipeptide, a histone deacetylase inhibitor, interrupts the association of AML1‐ETO with HSP90 and induces the proteasomal degradation of AML1‐ETO.^30^ Furthermore, our results also demonstrate that honokiol induces the degradation of AML1‐ETO via increasing the UbcH8, an E2‐conjugase.^10^ However, because AML1‐ETO alone is essential but not sufficient for the leukemic transformation, additional gene mutations, such as *c‐Kit*, are required for the leukaemia transformation in murine model. Thus, combination treatments targeting RUNX1/ETO and the frequent mutated proteins have achieved pre‐clinical efficiency. For example, oridonin and homoharringtonine exert synergistic effects against t (8; 21) leukaemia through targeting AML1‐ETO and c‐Kit mutation.^38^ However, the five‐year overall survival in AML patients with AML1‐ETO is still less than 30%. Recently, more studies focus the target geness which are regulated by AML1‐ETO and are required for leukemogenesis. For example, *COX2* 9 and *CCND2* 39 are two target genes by AML1‐ETO. Targeting COX2 and CCND2 by approved drugs inhibits the self‐renewal of LSCs and demonstrates preclinical therapeutic effects. Thus, specific AML1‐ETO‐targeting strategies will finally improve the overall survival in AML patients with AML1‐ETO.

As documented, AML1‐ETO protein can be degraded or cleaved by caspase‐3 in an apoptotic condition.^17^, ^40^ For example, eriocalyxin B and oridonin induce caspase‐3‐dependent apoptosis and degradation of AML1‐ETO. Inhibition of caspase‐3 activation by specific inhibitor effectively blocks eriocalyxin B‐ and oridonin‐induced degradation of AML1‐ETO protein.^41^, ^42^ Furthermore, activated caspase‐3 cleaves AML1‐ETO protein to four fragments of 70, 49, 40 and 25 kDa during apoptosis.^17^ These results suggest that caspase‐3 plays an important role in the degradation or cleavage of AML1‐ETO. Although MLT induces a significant increase in caspase‐3 activity in HL‐60 cells,^15^, ^35^ our results indicate that MLT treatment fails to markedly induce the activation of caspase‐3 in Kasumi‐1 and U937T cells. We speculate that MLT might induce the activation of different caspase family members. Most importantly, specific caspase‐3 inhibitor and pan‐caspase inhibitor do not block MLT‐induced inhibition of AML1‐ETO protein. Thus, our results suggest that MLT‐induced degradation of AML1‐ETO is independent of activated caspase‐3. Furthermore, knockdown of *AML1‐ETO* by ribozyme‐mediated targeting of fusion gene results in apoptosis or cell death.^43^ Therefore, we speculate that MLT‐induced degradation of AML1‐ETO leads to the apoptosis or cell death. However, we do not completely exclude the possibility that some unhealthy and dead cells show the lower levels of AML1‐ETO protein.MLT has been reported to modulate the ubiquitin‐proteasome system,^44^ which is the main pathway for the degradation of proteins. For example, MLT increases the transcription of the ubiquitin‐activating enzyme, E1, as well as transcription of the ubiquitin ligase, E3.^45^ Our previous study indicates that honokiol, a natural phenolic compound isolated from the plant Magnolia officinalis, induces proteasomal degradation of AML1‐ETO oncoprotein via increasing the expression of E2‐ubiquitin conjugase UbcH8 in leukaemia cells, indicating that AML1‐ETO protein is degraded through the UbcH8‐mediated ubiquitin‐proteasome system. In addition, E3‐ligase SIAH‐1 is mediated in the degradation of AML1‐ETO protein.^27^ Thus, AML1‐ETO protein can be degraded by ubiquitin proteasome system including UbcH8 and SIAH‐1. However, our results indicate that MG132, a prototypical proteasome inhibitor, fails to block MLT‐induced degradation of AML1‐ETO. Moreover, MLT does not increase the expression of UbcH8 and SIAH‐1 (data not shown). Therefore, MLT‐induced degradation of AML1‐ETO protein might be independent of the ubiquitin‐proteasome system.

Although inhibition of MT1/2 by a competitive antagonist luzindole did not prevent the degradation of AML1‐ETO by MLT, the utilized concentration of luzindole (5 µmol/L) is considerably lower than the utilized concentration of MLT (1 mmol/L). Therefore, luzindole might be outnumbered by MLT. Furthermore, in addition to MT1/2, the quinone reductase enzyme family (MT3) is the natural receptor by MLT.^46^ It is possible that MLT exerts its function via binding to its natural receptor MT3. In conclusion, we do not exclude the possibility that MLT degrades AML1‐ETO protein through binding to its natural receptor. Several articles demonstrate that MLT reduces the side‐effects of clinical drugs. For example, MLT alleviates the side‐effects of chemotherapy in breast cancer^47^ and metabolic side‐effects of olanzapine in schizophrenia.^48^ Furthermore, MLT as a dietary supplement has been used to improve the night time sleep for many years. No obvious side‐effects of MLT as a dietary supplement for people are reported. Most importantly, our results demonstrate that MLT has little effects on the colony formation in normal human and murine HSPCs. However, whether long‐term MLT treatment results in drug dependence and endocrine dyscrasia should be investigated.

The main shortfall of this study is that MLT decreases AML1‐ETO in high concentration (mM). This high concentration is not easily achieved under normal physiological conditions. Thus, nanoparticle‐based drug delivery should be used to maintain release, decrease the dose of MLT, and enhance treatment efficiency.^49^ In addition, although MLT presents anti‐LSC activity in leukaemia cells carrying AML1‐ETO, whether MLT inhibits self‐renewal of LSC in leukaemia without AML1‐ETO is still unknown. Therefore, MLL‐AF9‐induced murine leukaemia is required for the further study.^50^

Elimination of the initiating event in leukemogenesis has proved to be an extremely effective therapeutic strategy, such as Gleevec for BCR‐ABL in chronical myeloid leukaemia^51^ and all‐trans retinoic acid for AML with PML‐RARα.^52^ These therapeutic strategies have produced tremendous advances in the treatment of AML patients. However, at present no drugs for specially targeting AML1‐ETO fusion protein are used in clinical treatment. Our study indicates that MLT rapidly inhibits AML1‐ETO protein through up‐regulation of miR‐193a. Moreover, MLT presents strong anti‐LSC activity in AML cells with AML1‐ETO in vitro and in vivo. Considering that MLT is an endogenous molecule with low toxicity and favourable bio‐compatibility, MLT in combination with standard chemotherapy or radiotherapy might enhance the clinical outcome in t (8;21) AML patients.

## CONFLICT OF INTEREST

The authors of this manuscript have no conflicts of interest to disclose.

## AUTHOR CONTRIBUTIONS

ZB and YHG contributed to western blot, clinical samples collection, qRT‐PCR, flowcytometry assay, construction of plasmids and virus package. XCY, LB and LHY contributed to clinical samples collection, detection of luciferase, limiting dilution assay, cell sorting and mRNA extraction. CLL carried out cell culture and western blot. HXZ and WYF carried out mouse breeding, transplantation of murine leukaemia blasts, colony formation counting and isolation of murine leukaemia blasts. GSM performed the study design, statistical analysis and manuscript writing. This manuscript is not under review elsewhere and all authors read and approved the final manuscript.
