# Supplementary material for: Targeting miR‐193a‐AML1‐ETO‐β‐catenin axis by melatonin suppresses the self‐renewal of leukaemia stem cells in leukaemia with t (8;21) translocation
Source: J Cell Mol Med. 2019 May 22;23(8):5246–58. doi: 10.1111/jcmm.14399 (PMC6653044; doi:10.1111/jcmm.14399)
Supplement: Supplementary file 1 [file JCMM-23-5246-s001.docx]

**Table 1: The sequences of primers for qRT-PCR and construction of plasmids**

| **Primer** | **Sequence (5'-3')** |  |
| --- | --- | --- |
| Bcl-2-L | ATC CAG GAT AAC GGA GGC TG |  |
| Bcl-2-R | GAA ATC AAA CAG AGG CCG CA |  |
| GCSFR-L | acc tct cct gcc tca tga ac |  |
| GCSFR-R | cag agt gaa gct ggt ggg ta |  |
| MPO-L | tga tcg gtt ttg gtg gga ga |  |
| MPO-R | atg atc cgg ggc aat gag at |  |
| GMCSF-L | act tcc tgt gca acc cag at |  |
| GMCSF-R | cca gca gtc aaa ggg gat ga |  |
| GAPDH-L | GGT CGG AGT CAA CGG ATT TG |  |
| GAPDH-R | ATG AGC CCC AGC CTT CTC CAT |  |
| MT1-L | TCA ACC GCT ACT GCT ACA TC |  |
| MT1-R | TCA TCA GTG GAG ACG GTT TC |  |
| MT2-L | TCA TCG GCT CTG TCT TCA ATA |  |
| MT2-R | ACT GGG TGC TGG CGG TCT GGA |  |
| LVX-miR-193a-L | GGA ATT CCG AGC GTC GTG TAA CCC TTG |  |
| LVX-miR-193a-R | GAC TAG TCG AGC GCA CCT CAC CAC TC |  |
| LVX-β-catenin-L | GCT CTA GAG CAT GGC TAC TCA AGC TGA TTT |  |
| LVX-β-catenin-R | CGG GAT CCC GTT ACA GGT CAG TAT CAA ACC |  |
