# Supplementary figures and images for: Targeting miR‐193a‐AML1‐ETO‐β‐catenin axis by melatonin suppresses the self‐renewal of leukaemia stem cells in leukaemia with t (8;21) translocation
Source: J Cell Mol Med. 2019 May 22;23(8):5246–58. doi: 10.1111/jcmm.14399 (PMC6653044; doi:10.1111/jcmm.14399)

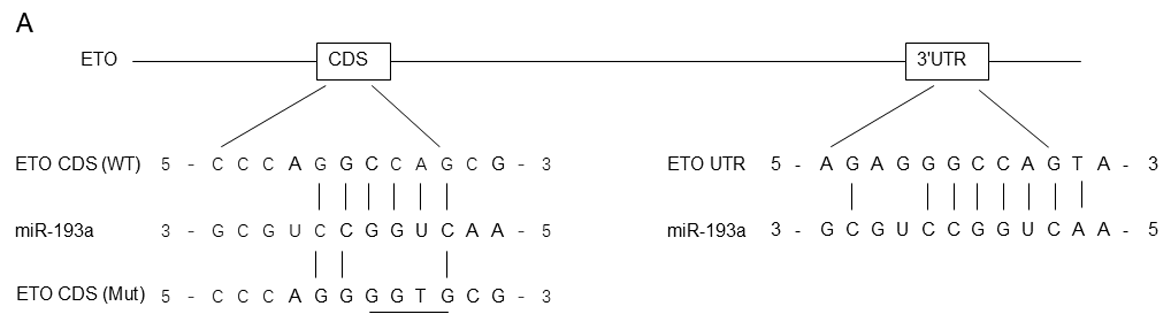

Supplement: Supplementary file 2 [file JCMM-23-5246-s002.tif]

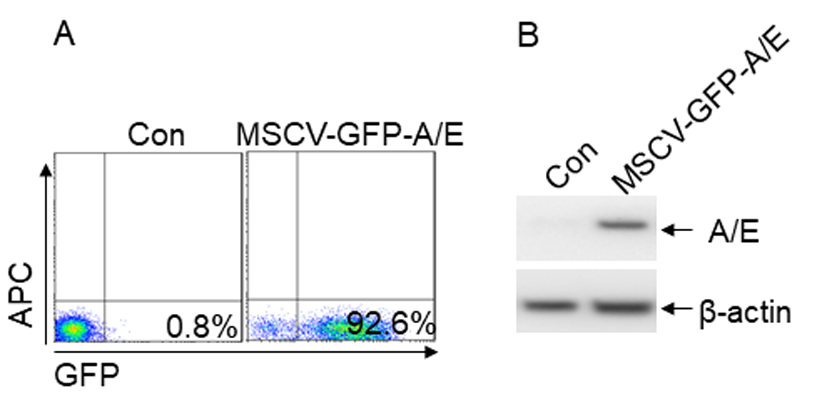

Supplement: Supplementary file 3 [file JCMM-23-5246-s003.tif]

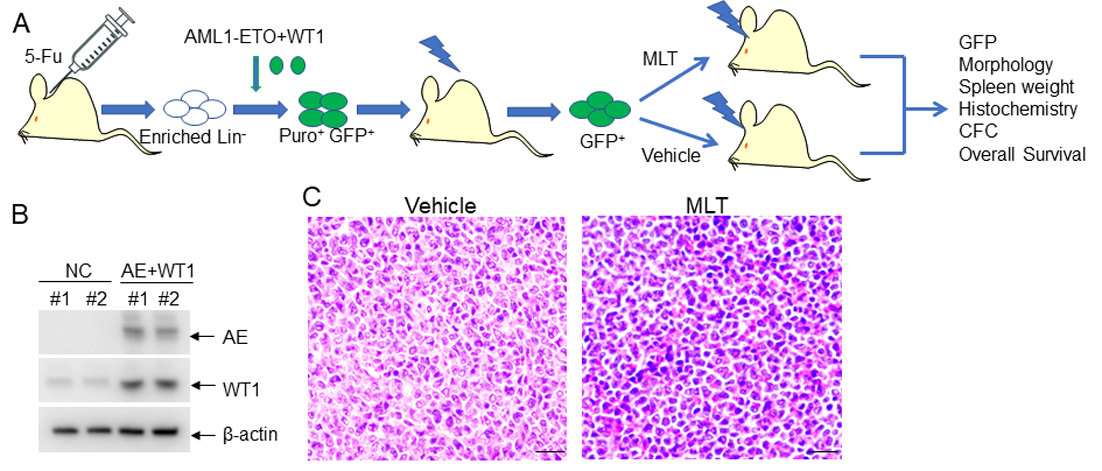

Supplement: Supplementary file 4 [file JCMM-23-5246-s004.tif]

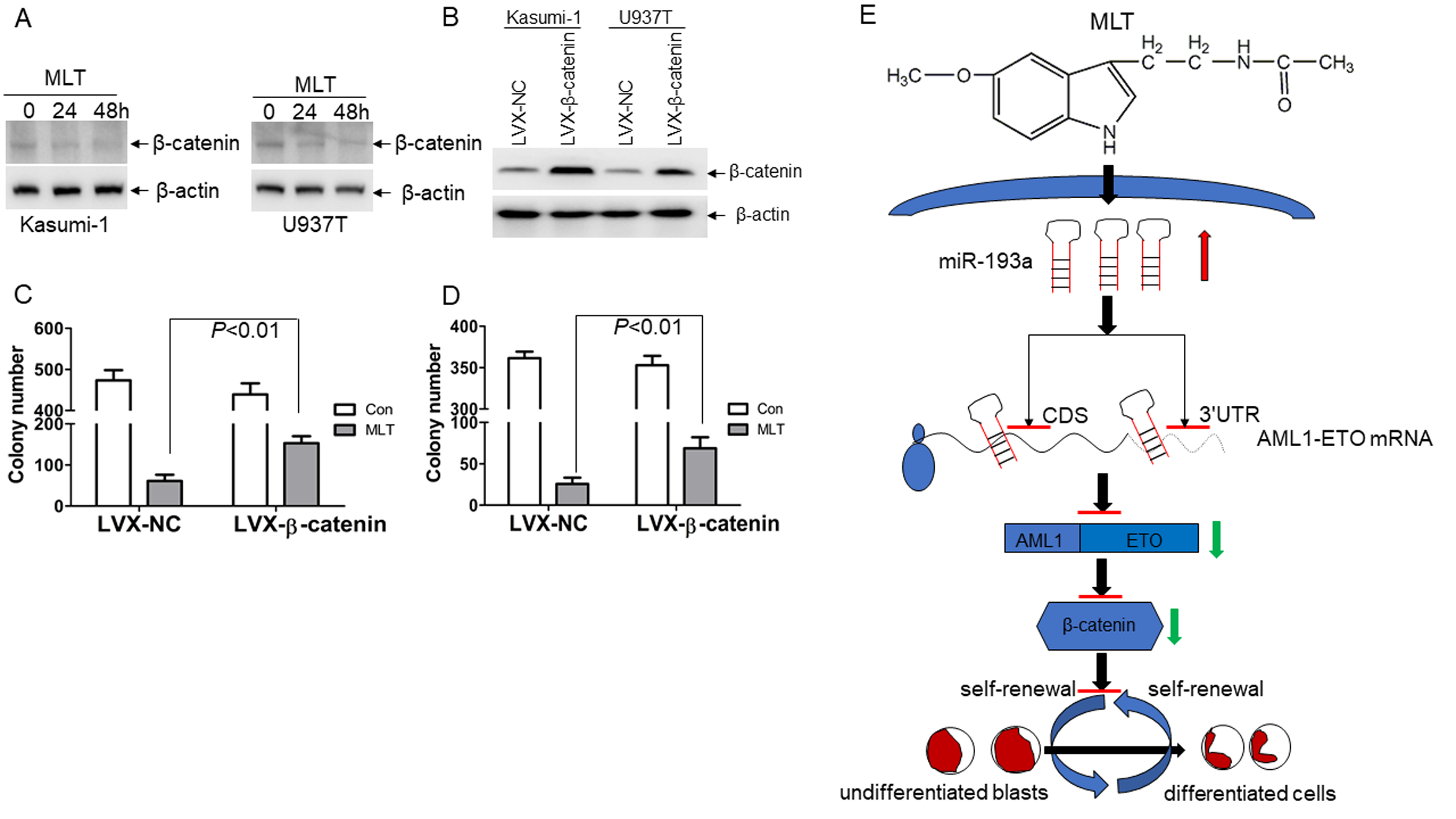

Supplement: Supplementary file 5 [file JCMM-23-5246-s005.tif]

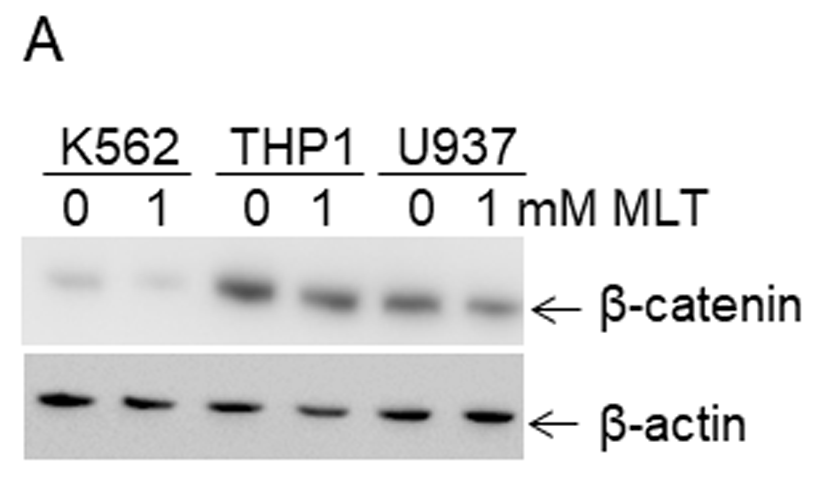

Supplement: Supplementary file 6 [file JCMM-23-5246-s006.tif]
