# Supplementary material for: Targeting miR‐193a‐AML1‐ETO‐β‐catenin axis by melatonin suppresses the self‐renewal of leukaemia stem cells in leukaemia with t (8;21) translocation
Source: J Cell Mol Med. 2019 May 22;23(8):5246–58. doi: 10.1111/jcmm.14399 (PMC6653044; doi:10.1111/jcmm.14399)
Supplement: Supplementary file 7 [file JCMM-23-5246-s007.docx]

Supplemental Figure legend

Fig S1: (A) Schematic of putative binding sites for miR-193a in CDS and 3'UTR of *ETO*.

Fig S2: Overexpression of AML1-ETO in U937 cells. (A) U937 cells were transduced with or without MSCV-GFP-IRES-AML1-ETO. GFP^+^ cells were measured by flowcytometry. (B) Western blot was detected in U937 cells transduced with or without MSCV-GFP-IRES-AML1-ETO.

Fig S3: Anti-leukemia activity of MLT in AML1-ETO-induced leukemia mice. (A) A schematic outline of the *in vivo* experiment using AML1-ETO plus WT1-induced leukemia mice model. (B) The protein expressions of AML1-ETO and WT1 were detected in BM cells from two normal control mice or AML1-ETO plus WT1-induced leukemia mice. (C) HE staining of spleen from AML1-ETO plus WT1-induced leukemia mice treated with or without MLT.

Fig S4: β-catenin mediates MLT-induced anti-leukemogenesis and an illustration of MLT-induced anti-self-renewal capacity of LSC through miR-193a-AML1-ETO-β-catenin axis. (A) The protein expression of β-catenin was detected in Kasumi-1 and U937T cells treated with 1 mM MLT for 24 and 48 h. (B) The protein expression of β-catenin was detected in Kasumi-1 and U937T cells, which were transduced with LVX-NC or LVX-β-catenin ovexpressing β-catenin. (C and D) Colony formation was counted in LVX-NC- or LVX-β-catenin-transduced Kasumi-1 (C) and U937T cells (D), which were treated with or without MLT. (E) An illustration of potential anti-self-renewal activity of LSC by MLT through miR-193a-AML1-ETO-β-catenin signal pathway in acute myeloid leukemia with t (8;21) translocation. MLT increases the expression of miR-193a in leukemia cells. The increased expression of miR-193a decreases AML1-ETO expression via binding its CDS and 3'UTR sites. Thus, MLT induces the degradation of AML1-ETO oncoprotein through increasing the expression of miR-193a. The downregulation of AML1-ETO decreases the expression of β-catenin, which inhibits the self-renewal of LSC and enhances the differentiation. Therefore, MLT presents anti-leukemogenesis through miR-193a-AML1-ETO-β-catenin axis.

Fig S5: MLT slightly decreases the expressions of β-catenin in leukemia cells with negative expression of AML1-ETO. (A) The expressions of β-catenin were measured by western blot in K562, THP1, and U937 cells treated with MLT (1 mM) or not for 24 h.
